# Friendships Forged in Fitness: An Ethnographic Exploration of Older Women’s Social Experiences in Water Aerobics

**DOI:** 10.1093/geroni/igaa057.1522

**Published:** 2020-12-16

**Authors:** Britteny Howell, Samantha Wanner

**Affiliations:** University of Alaska Anchorage, Anchorage, Alaska, United States

## Abstract

Research shows that participants, especially older women, are more likely to adhere to a fitness program when they have social supports. Gerontology research also demonstrates that the social relationships forged by older women at community and fitness centers can be long-lasting and provide a variety of supportive functions. Older adults respond well to pool- or water-based aerobic exercises that are safe on the joints and provide a comfortable environment away from the intimidating nature of the gym. Therefore, water-based classes provided at community fitness centers are well positioned to provide ample social opportunities to further reinforce continued physical activity for older women, resulting in health and quality-of-life improvements. This project is a 5-month ethnographic exploration of the social relationships created and maintained in the context of water-based fitness classes (water aerobics) at a local community center (YMCA) that is attended by a culturally diverse group of older adults. The friendships forged by women in the pool at the YMCA provide a variety of social supports that help to maintain healthy aging outcomes among participants. Drawing on components of Activity Theory and Social Support Theory, this presentation utilizes participant observation, semi-structured interviews, and questionnaires (N=35) to provide an anthropological “thick description” of the important role that fitness center friendships can form in the social lives of older women in the U.S.

